# Severe menstrual pain is not normal: adolescent dysmenorrhoea as a marker of future chronic pain

**DOI:** 10.61622/rbgo/2026rbgoedt2

**Published:** 2026-05-12

**Authors:** Omero Benedicto Poli-Neto, Rachel Reid-McCann, Katy Vincent

**Affiliations:** 1 Universidade de São Paulo Department of Obstetrics and Gynecology Ribeirão Preto SP Brazil Department of Obstetrics and Gynecology, Universidade de São Paulo, Ribeirão Preto, SP, Brazil.; 2 Universidade de São Paulo Laboratory for Translational Data Science Ribeirão Preto SP Brazil Laboratory for Translational Data Science, Universidade de São Paulo, Ribeirão Preto, SP, Brazil.; 3 University of Oxford Nuffield Department of Women's and Reproductive Health UK Nuffield Department of Women's and Reproductive Health, University of Oxford, UK.

Menstruation is a natural and universal occurrence shared by most individuals assigned female at birth, spanning through menarche to the menopause. However, the association of this natural phenomenon with significant physical and emotional discomfort has been historically neglected.^( 1 )^ Pain associated with menstruation, or dysmenorrhoea, is among the most common gynaecological complaints, affecting between 45% and 95% of menstruating women.^( 2 )^ About 30% of these women complain of suffering so seriously with menstrual pain that it impacts daily activities such as learning or occupational responsibilities.^( 3 , 4 )^

Despite the high prevalence and substantial negative impact, dysmenorrhoea remains largely normalized, underrecognized, and undertreated.^( 5 )^ Many young women and adolescents endure debilitating pain monthly without seeking medical attention, believing it to be the "natural" and inevitable companion of menstruation.^( 6 )^ This culturally internalized assumption promotes underreporting, delaying treatment and diagnosis, and the broader delegitimization of female pain.^( 7 )^ Even in healthcare settings, menstrual pain is often minimised, solidifying stigma and propagating the broader silent suffering cycle.

Over the years, increasing evidence has revealed that dysmenorrhoea is not just a short-lived issue limited to the menstrual cycle, but that it could also be an early predictor of susceptibility to chronic pain. Among the most recent contributions to this area is the recent UK-based cohort study published in the Lancet Child & Adolescent Health.^( 8 )^

Using data from the Avon Longitudinal Study of Parents and Children (ALSPAC), we examined 1,157 participants, assessing menstrual pain at 15 years and the presence of chronic pain at 26 years of age. We found that the severity of dysmenorrhoea during adolescence predicted the later life risk of chronic pain, even after controlling for body mass index, adverse childhood experiences, premenarchal depressive symptoms, and maternal education.

Compared with adolescents without menstrual pain, those with mild dysmenorrhoea had an adjusted relative risk of 1.23 (95% CI 0.85–1.74, p=0.270) for developing chronic pain later in life. The risk increased to 1.65 (1.22–2.18, p=0.002) for those with moderate pain (painful enough that person could not easily forget about them, whatever was going on) and 1.76 (1.23–2.39, p=0.003) for those with severe pain (so that person were unable to continue with normal activities). Evidence of a definite linear dose-response relationship was observed, showing an estimated 25% increase in chronic pain risk for each unit-increase in the severity of dysmenorrhoea.

Teenagers experiencing severe dysmenorrhoea were more likely to experience pain at sites throughout the body (so not just limited to the pelvis), most notably in the abdomen, lower back, and head. Of interest in this study is the observation that anxiety and depressive symptoms only explained a small percentage of this association, suggesting that neurobiological mechanisms potentially including central sensitisation might play a more prominent role than purely psychological factors.

These results echo previous studies demonstrating increased pain sensitivity in women with dysmenorrhoea,^( 9 - 12 )^ functional and morphological alterations in the brain,^( 13 - 17 )^ and dysregulation of neuroendocrine axes.^( 18 )^ These findings, as a whole, provide evidence to support the hypothesis that adolescents experiencing recurrent episodes of intense menstrual pain are predisposed to developing chronic pain later in life, due to "imprinting" of the malleable adolescent nervous system and thus heightened sensitivity to future stimuli. This is plausible considering adolescence is a developmental stage of heightened plasticity of the nervous system.

The effects of severe dysmenorrhoea transcend the mere physical domain. Debilitating menstrual discomfort has been linked to diminished social interactions,^( 19 )^ reduced academic outcomes, increased rates of absenteeism, and lower levels of concentration.^( 20 , 21 )^ Students suffering from dysmenorrhoea are as much as eight times more predisposed to encounter hindered academic success.^( 2 )^

Menstrual pain, from the psychosocial perspective, prevents engagement in physical and recreational activities, restricts interaction with the social network, damages self-esteem, and contributes to anxiety, depression, and chronic fatigue.^( 22 , 23 )^ In Brazil, as in much of the world, where the mean age at menarche is around 12-14 years, the beginning of menstrual pain happens just at the time of the most significant developmental and identity-forming stage of life. Repeated and frequently disregarded pain, endured at this stage of life, is likely to have a long-lasting impact on the emotional and developmental trajectories of adolescents. It must therefore not only be considered an occasional annoyance, but also as a major public health concern, and one which has a far-reaching impact upon productivity, education, mental health, and equity between the sexes.

Dysmenorrhoea deserves inclusion in primary prevention strategies and early management of chronic pain. Young adolescents have long been ill-equipped for puberty and menstruation.^( 24 )^ Adolescent health programmes have rarely included menstrual education as a structured component, despite the fact that much could be learned through this.^( 25 )^

Gynaecologists and primary healthcare providers must acknowledge and manage menstrual pain as an authentic clinical syndrome and not merely as an inconspicuous grievance or a "natural penalty" of femininity. Appropriate management, pharmacological or otherwise, should be implemented to alleviate intermittent acute pain and, potentially, to reduce the risk of its transition to chronicity. Non-steroidal anti-inflammatory drugs^( 26 )^ (NSAIDs) and hormonal contraceptives^( 27 )^ remain first-line options, but complementary approaches such as regular physical exercise^( 28 )^ and psychological interventions,^( 29 )^ among others, have demonstrated their efficacy and should be recommended promptly. Although young people do engage with self-management strategies (including over the counter analgesia and non-pharmacological approaches) this frequently does not provide adequate relief and there is little information available to guide their choices.^( 30 )^

Diagnosis is essentially clinical, and often the first care can often begin without additional investigations. Education, reassurance, and guidance regarding helpful measures of self-care, such as warmth, transcutaneous electrical nerve stimulation (TENS), exercise, or rest, should be integrated into a trial of pharmacologic therapy as needed, usually NSAIDs and/or hormones, and followed up on if symptoms persist. However, certain parameters would be considered reason for suspicion of an underlying or associated disease and require investigations, such as congenital anomalies of the genitourinary tract, severe and progressive pain since menarche, familial history, abnormal bleeding or discharge, failure of empirical treatment by hormones or NSAIDs, patient or physician concern, or abnormal physical examination findings. Under these conditions, a referral for a pelvis ultrasound and evaluation is warranted, depending on local facilities available.^( 31 )^ Despite investigations into potential systemic and menstrual effluent biomarkers by a number of researchers, no study has as yet met standards of sensitivity or specificity for clinical utility.^( 32 )^

Beyond individual care, there is also the crucial educational and cultural dimension. Awareness of menstruation specifically for adolescents, families, education providers, and healthcare providers, serves as a key baseline for breaking the silence and stigma that still shrouds menstruation. Studies show that many young people conceal their distress because they feel ashamed or because they believe pain goes along with femininity.^( 33 )^ This misconception prevents timely treatment (including for conditions underlying some cases of dysmenorrhoea, for example endometriosis), weakens the doctor-patient relationship, and perpetuates gender inequities in access to compassionate and effective care.

Legitimising menstrual pain involves the recognition that menstrual symptoms are genuinely experienced by menstruators and are worthy of attention, even where demonstrable pathologies such as endometriosis or adenomyosis are not present. For so long, menstrual suffering has been accounted for by skewed perspectives, as exaggeration, "hysteria", or weakness. That gender bias exists even now, albeit often implicitly, and gives rise to rushed consultations, inadequate investigation, superficial advice, and unfairness.^( 34 )^

Contemporary evidence-based medicine strongly supports the stance that severe dysmenorrhoea foreshadows subsequent chronic pain and reflects how our societies either address or neglect women's pain. An integration of caring, evidence-based, and educated care, as well as translational studies, can significantly shift this present paradigm. It is crucial to include menstrual pain issues within school health policy. School education programs concerning reproductive health should cover this topic comprehensively and openly, giving due respect to the varied experiences, including those of transgender and non-binary students, and facilitating early access to proper medical care. An awareness of the varied aspects of the impact of dysmenorrhoea represents, ultimately, an action towards the promotion of equity and citizenship in the health field.

Although the association between dysmenorrhoea and chronic pain is now well established, the underlying neurobiological and genetic mechanisms have yet to be clearly elaborated. It would also be of key importance to investigate the question of whether early treatments of dysmenorrhoea would be an efficacious primary prevention of chronic pain. Randomised clinical trials and population-based studies of wide generalisability and large sample size would be of primary importance to answer this question. Another pressing challenge is promoting greater inclusion of transmasculine and non-binary communities within menstrual health studies, given their unique experiences and the additional barriers to care that they experience.^( 35 )^ It is only by collecting truly representative data that the establishment of fair and inclusive public health policy becomes possible. Finally, most crucial is the fact that pain is a complex biopsychosocial experience. Addressing dysmenorrhoea effectively demands an interdisciplinary approach, including translational science, clinical practice, and societal transformation.

The latest findings in this research area bring forth an irrefutable message: severe menstrual pain during adolescence should not be considered normal and cannot be dismissed. Such a symptom constitutes an invaluable moment of decisive action for clinical, pedagogical, and societal practice. To face dysmenorrhoea with the seriousness it deserves goes far beyond just relieving a monthly symptom. It represents an investment in the future prevention of chronic pain, the betterment of mental health, and the quality of life of future generations. It also represents an equity statement, acknowledging the pain borne by women and confronting the centuries of disregard and silence.

Gynaecologists, teachers, health managers, and policy leaders have an essential duty. It is time to listen to young people respectfully and conscientiously, promote empowering menstrual education, and to ensure universal access to evidence-based care. In doing so, we shall transform what once went underappreciated into the essence of integrated health. Severe menstrual pain is not normal. It is an early warning, and an opportunity to provide better care.
